# The mediating factors in the relationship between lower urinary tract symptoms and health-related quality of life

**DOI:** 10.1186/s13104-017-2928-7

**Published:** 2017-11-23

**Authors:** Weng-Yee Chin, Edmond P. H. Choi, Eric Y. F. Wan, Cindy L. K. Lam

**Affiliations:** 1 Department of Family Medicine and Primary Care, University of Hong Kong, 3/F., 161 Main Street, Ap Lei Chau Clinic, Ap Lei Chau, Hong Kong; 2 School of Nursing, University of Hong Kong, Pokfulam, Hong Kong

**Keywords:** Anxiety, Depression, Lower urinary tract symptoms, Mental health, Nursing, Primary care, Quality of life, Stress

## Abstract

**Objectives:**

An earlier study found that mental health partially mediates the relationship between lower urinary tract symptoms (LUTS) severity and health-related quality of life (HRQOL). In other words, LUTS adversely affects mental health, which in turn adversely affects HRQOL. A major limitation of the previous study was its cross-sectional design. The aim of this study is to evaluate whether changes in mental health mediated the association between changes in the severity of LUTS and changes in HRQOL over 24 months by using Baron and Kenny’s regression procedure and Preacher and Hayes’s bootstrapping method.

**Results:**

We found that changes in mental health were a mediator in the relationship between the change of LUTS severity and the change of LUTS-specific HRQOL. Changes in LUTS severity lead to changes in mental health, which in turn affects the change of LUTS-specific HRQOL. It was observed however that changes in mental health did not mediate the relationship between the change of LUTS severity and the change of the physical aspects of generic HRQOL. These findings suggest that in order to optimize LUTS-specific HRQOL, both LUTS severity and mental health may need to be addressed concurrently.

## Background

Although lower urinary tract symptoms (LUTS) are not a major cause of mortality, they are highly prevalent chronic conditions [1] which negatively affects mental health [2, 3] and health-related quality of life (HRQOL) [4]. Due to the chronic and non-fatal nature of LUTS, most patients have to learn to live with their urinary problems. Therefore, improving HRQOL are often the key goals of most LUTS interventions.

### Mental health as a mediator in the relationship between symptom severity and HRQOL

A mediation model can be used to explain the mechanism that underlies an observed relationship between an independent variable and a dependent variable via the inclusion of a third variable, as known as a mediator. Rather than a direct causal relationship between an independent variable and a dependent variable, a mediation model proposes that an independent variable produces an intermediate effect (a mediator), which in turn influences the dependent variable [5, 6]. Exploring mediators is important in prevention and treatment research because interventions can target treatment to address the mediating factors to help improve clinical outcomes.

A previous study found that mental health is a mediating factor in the relationship between LUTS severity and HRQOL. In other words, LUTS severity affects mental health, which in turn impairs HRQOL [7]. One major limitation of that study was its cross-sectional design. To strength the evidence, this study aims to evaluate if changes in mental health mediate the relationship between change in LUTS severity and change in HRQOL using longitudinal data. The study’s hypothesis is that changes in LUTS severity leads to changes in mental health, which in turn lead to changes in HRQOL.

## Methods

This is a secondary analysis using the data from a 2-year prospective longitudinal health services study evaluating the quality of care of nurse-led care for patients with LUTS in Hong Kong. The details of the primary study have been previously reported [8].

All field workers received training on subject recruitment procedures, consent and documentation. Patients were recruited from clinics in person by field workers and invited to join the study. Subjects were excluded if they did not have LUTS, were not able to give consent, could not understand Chinese or were younger than 18 years. Recruited subjects were either under usual care or under nurse-led care for their LUTS problems. Subjects who consented to join the study provided their telephone numbers and were subsequently contacted by trained interviewers. A structured questionnaire was administered by telephone within 2 weeks of recruitment (baseline interview) and at 24-month (follow-up interview). Before starting the telephone interviews, interviewers asked the name of respondents to ensure that they were actually speaking with the patient. To maintain the data quality, the standardised script and questionnaires were read verbatim in order to standardize the interviews.

## Study instruments

### The International prostate symptom score (IPSS)

The IPSS was used to assess LUTS severity, with a high score indicating more severe LUTS. The psychometric properties of the IPSS (administered on telephone interviews) have been confirmed in male and female patients in Chinese primary care [9, 10].

### Modified Incontinence Impact Questionnaire-Short Form (IIQ-7)

The modified IIQ-7 was used to evaluate LUTS-specific HRQOL with a higher score indicating more negative impacts of LUTS on HRQOL. The psychometric properties of the modified IIQ-7 (administered on telephone interviews) have been validated in male and female patients in Chinese primary care [9, 11].

### The Short Form-12 Health Survey Version 2 (SF-12 v2)

The SF-12 v2 was used to evaluate the generic HRQOL. The instrument can generate two distinct summary scores, namely the physical (PCS) and mental component summary (MCS) scores. A higher score of the SF-12 v2 indicates better generic HRQOL. The SF-12 v2 has been validated and normed on the Hong Kong general population [12, 13].

### Depression Anxiety and Stress Scale-21 (DASS-21)

The DASS-21 consists of three subscales to assess depressive, anxiety and stress symptomology. Each subscale consists of seven questions, with higher scores indicting poorer mental health [14]. The psychometric properties including validity, reliability and responsiveness of the DASS-21 have been confirmed [9, 15].

## Statistical analysis

The independent variable was the change of LUTS severity as measured by the IPSS. The mediator was the change of mental health as assessed by the DASS-21. The dependent variables were the change of HRQOL as assessed by the modified IIQ-7 and the SF-12 v2 PCS. We deliberately did not put the SF-12 v2 MCS in the mediation model because the DASS-21 and SF-12 v2 MCS measure a very similar construct.

Two approaches were used to test the hypothesis. First, Baron and Kenney’s multistage regression approach [16] was used to explore the relationship between the change of LUTS severity, the change of mental health and the change of HRQOL.

1. A simple regression analysis with the change of LUTS severity as assessed by the IPSS (independent variable) predicting the change of HRQOL as assessed the modified IIQ-7 and SF-12 v2 PCS (dependent variable) was conducted, respectively.
2. The change of the DASS-21 scores including depression, anxiety and stress scores (mediator) was each regressed on the change of LUTS severity.
3. The change of HRQOL was regressed on, the change of depression, anxiety and stress scores, respectively.
4. The change of HRQOL was regressed on both, the change of depression, anxiety and stress scores and the change of LUTS severity, accordingly.

Second, Preacher and Hayes’s bootstrapping method [17] was used to estimate the indirect effect (the change of LUTS severity affects the change of mental health, which in turn affects the change of HRQOL). This method is considered to be more powerful than the Sobel test [18]. Point estimates were based on a 5000 bootstrapping sample. Besides, 95% confidence intervals were constructed. An indirect effect was considered statistically significant if the confidence interval did not contain zero.

**Fig. 1:**
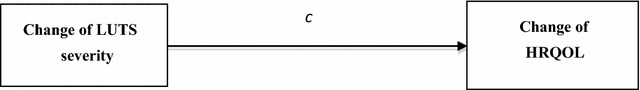
The total effect of lower urinary tract symptoms severity on health-related quality of life

**Fig. 2:**
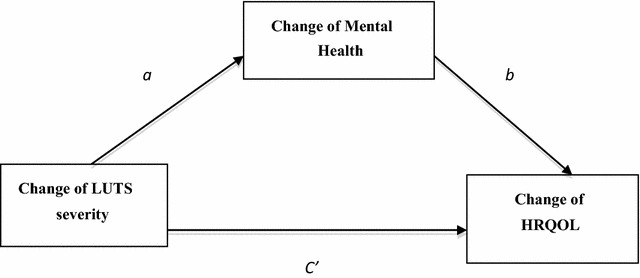
Hypothesized mediation model. It was hypothesized that the change of mental health mediated the relationship between the change of lower urinary tract symptoms severity and the change of health-related quality of life

The proposed mediation model is shown in Figs. 1 and 2. Figure 1 shows the effect of the change in LUTS severity on the change in HRQOL, without considering any mediators. It was a total effect. In Fig. 2, *a* is the coefficient for the change in LUTS severity in the model predicting the change of mental health, and *b* and *c’* are the coefficients in the model predicting the change of HRQOL from the change in mental health and the change in LUTS severity, respectively.

*c’* quantifies the direct effect of the change in LUTS severity on the change in HRQOL. The product of *a* and *b* (point estimate) quantifies the indirect effect of the change in LUTS severity on the change in HRQOL through mental health.

Age, gender, education status, marital status and working status were all adjusted in the mediation analysis.

The ratio of the indirect effect to the total effect was used to quantify the effect size of the mediation model as an indication of the proportion of the total effect mediated by the mediator [19].

All significance tests were two-tailed and findings with a p value less than 0.05 were considered statistically significant. SPSS Version 23.0 was used to perform all statistical analyses.

## Results

Initially, 720 subjects were recruited. 519 subjects completed the telephone interview at baseline (dropout rate: 29.2%). Of these, 335 subjects also completed the telephone interview at 24-months (dropout rate: 35.5%). Baseline characteristics of the study subjects are shown in Table 1.

**Table 1:** Baseline characteristics of subjects

	All subjects (N = 519)
Socio-demographic (%, n)	
Age (mean ± SD)	62.45 ± 10.95 (519)
Age group (years)	
18–65	61.08% (317)
> 65	38.92% (202)
Gender	
Female	55.88% (290)
Male	44.12% (229)
Marital status	
Not married	23.51% (122)
Married	76.49% (397)
Employment status	
Not working	65.51% (340)
Working	34.49% (179)
Household income	
< $20,000	73.19% (333)
≥ $20,000	26.81% (122)
Smoking status	
Non-smoker	96.96% (478)
Smoker	3.04% (15)
Drinking status	
Non-drinker	63.89% (315)
Drinker	36.11% (178)

### LUTS- specific HRQOL

Table 2 shows the results of the mediation models. After controlling for mental health, the direct effect of LUTS severity as measured by the IPSS on LUTS-specific HRQOL as measured by the IIQ-7 remained statistically significant but less than the total effect (β = 0.238).

**Table 2:** Coefficients of direct effect and total effect and the results of bootstrapping method

	Coefficient *a*	Coefficient *b*	Coefficient *c’* (direct effect)	Total effect *C*	Indirect effect		Bias corrected 95% CI		Effect size
					Point estimate	SE	Lower	Upper	
Depression									
IIQ-7	0.162* (0.082, 0.235)	0.304* (0.156, 0.456)	0.189* (0.105, 0.273)	0.238* (0.144, 0.326)	0.049	0.018	0.020	0.090	0.207
SF-12 v2 PCS	0.162* (0.078, 0.237)	− 0.312 (− 0.711, 0.090)	− 0.333* (− 0.568, − 0.093)	− 0.383* (− 0.613, − 0.133)	− 0.051	0.035	− 0.129	0.011	0.132
Anxiety									
IIQ-7	0.138* (0.078, 0.195)	0.289* (0.114, 0.503)	0.198* (0.116, 0.283)	0.238* (0.144, 0.326)	0.040	0.017	0.014	0.079	0.167
SF-12 v2 PCS	0.138* (0.079, 0.195)	− 0.288 (− 0.932, 0.297)	− 0.344* (− 0.596, − 0.109)	− 0.383* (− 0.613, − 0.133)	− 0.040	0.046	− 0.142	0.038	0.103
Stress									
IIQ-7	0.191* (0.134, 0.255)	0.219* (0.085, 0.386)	0.196* (0.109, 0.285)	0.238* (0.144, 0.326)	0.042	0.017	0.016	0.083	0.176
SF-12 v2 PCS	0.191* (0.135, 0.255)	− 0.140 (− 0.543, 0.287)	− 0.356* (− 0.609, − 0.107)	− 0.383* (− 0.613, − 0.133)	− 0.027	0.042	− 0.111	0.054	0.070

*IIQ-7* Incontinence Impact Questionnaire-7, *SE* standard error, *CI* confidence interval

* Significant with P < 0.05

- The regression coefficient of direct effect in the depression model: β = 0.189.
- The regression coefficient of direct effect in the anxiety model: β = 0.198.
- The regression coefficient of direct effect in the stress model: β = 0.196.

**Fig. 3:**
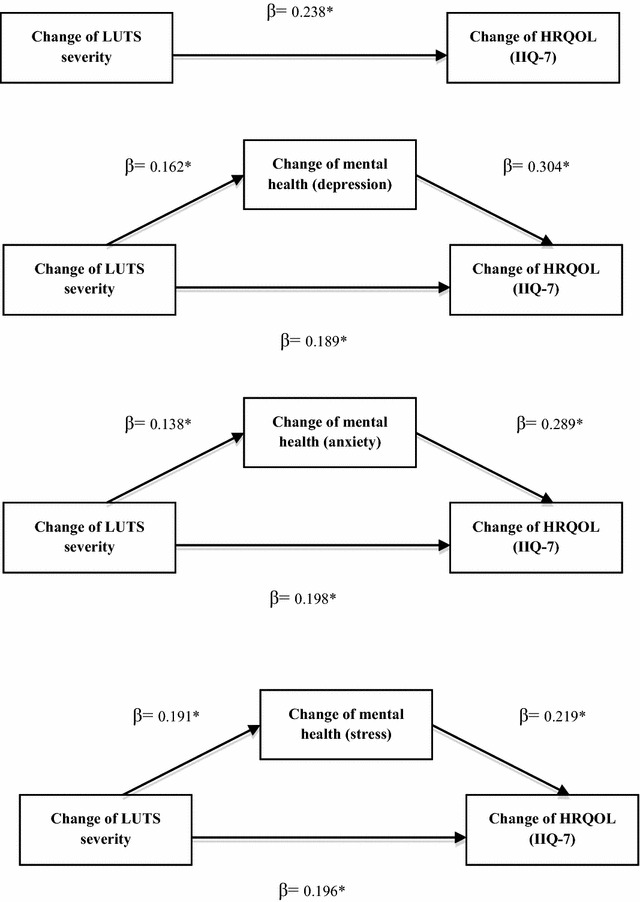
Mediation models (IIQ-7). The change of mental health mediated the change of lower urinary tract symptoms (LUTS) severity and the change of health-related quality of life (HRQOL) as measured by as measured by the condition-specific HRQOL measure (IIQ-7). The direct effects of LUTS severity on LUTS-specific HRQOL were statistically significant (through depression β = 0.189; through anxiety β = 0.198; through stress β = 0.196; P < 0.05) but less than the total effect (β = 0.238). *P < 0.05 β: the regression parameter estimate. The models were adjusted for socio-demographic factors

The bootstrapping method showed that the 95% confidence intervals (corrected for bias) did not contain zero, supporting partial mediation models. LUTS affected the LUTS-specific HRQOL both directly as well as through mental health. Figure 3 shows the results of the mediation model (IIQ-7 as the outcome). The effect sizes were 0.21, 0.17 and 0.18, suggesting that the indirect effect accounted for 21, 17 and 18% of the total effect in the depression, anxiety and stress models respectively.

### Generic HRQOL

**Fig. 4:**
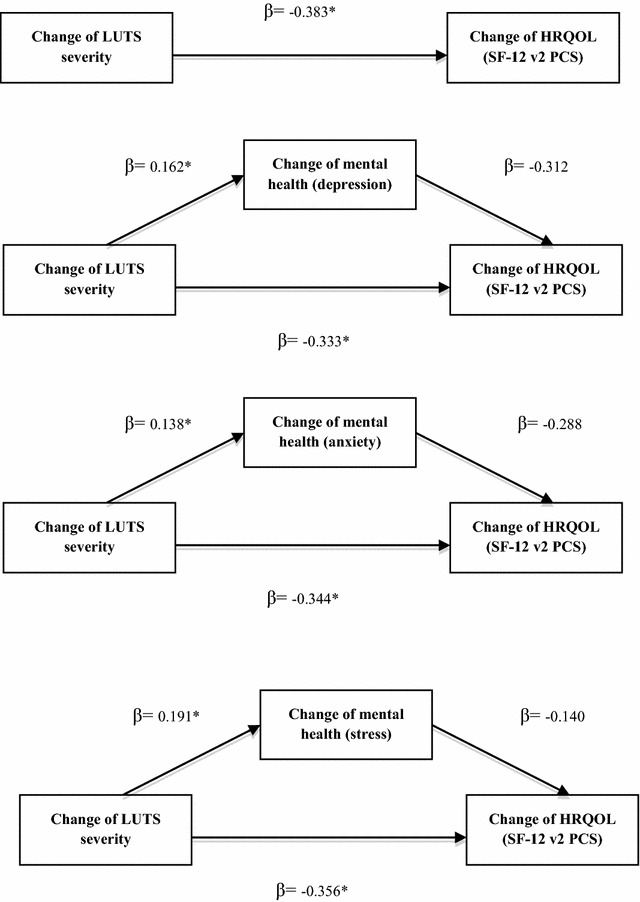
Mediation models (SF-12 v2 PCS). The change of mental health did not mediated the change of lower urinary tract symptoms severity and the change of health-related quality of life as measured by as measured by the physical component summary of Short-Form 12 Health Survey version 2 (SF-12 v2). *P < 0.05 β: the regression parameter estimate. The models were adjusted for socio-demographic factors

In the present study, the change of mental health was not a mediator between the change of LUTS severity and change in the physical component of generic HRQOL. Figure 4 shows the results of the mediation model (SF-12 v2 PCS as the outcome).

## Discussion

The findings of this present study strengthen the earlier study [7] in three ways. First, longitudinal data was used in the mediation analysis. Second, the previous study did not control for possible confounding variables in the mediation analysis. In the present study, we controlled for socio-demographic factors in the mediation analysis to provide a more precise estimate. Third, we provided the effect sizes of the mediating effect of different mediators.

### Key finding 1

We found that change in mental health partially mediated the relationship between the change in LUTS severity and change in LUTS-specific HRQOL. This means that changes in LUTS can lead to changes in LUTS-specific of HRQOL both directly and through changes in depressive, anxiety and stress symptoms. There are some possible explanations for this finding. First, LUTS can cause anxiety and embarrassment, may prevent patients from going out as much, and can have a negative impact on an individual’s sex life [20]. A previous qualitative study found that people with LUTS are often anxious about sudden urinary leakage in public situations and avoided going to other people’s houses. When they go out, they will seek out to use the toilets regardless of whether they needed to go or not [21]. Our mediation analysis further confirmed this link between symptom, anxiety and HRQOL (i.e. LUTS leads to anxiety, which in turn leads to more self-restriction on their daily life). Second, previous studies found that LUTS are significantly associated with sadness [22] and increased odds of having depressive symptoms [2]. LUTS patients with more severe depressive symptoms might have negative thinking and underestimate their ability to engage in daily activities, or exaggerate the negative impacts of LUTS on their daily lives, which can further impair their HRQOL [23, 24].

There are some clinical implications. First, given that the change of mental health partially mediates the association between the change of LUTS severity and the change of LUTS-specific HRQOL, interventions for LUTS patients may be most effective when they target alleviation of both the LUTS symptoms and psychological distress associated with LUTS. Second, the effect size of the mediating effect of depressive symptoms, anxiety and stress were quite similar, suggesting that these components of mental health should be addressed holistically in patients with LUTS.

### Key finding 2

The change of mental health did not have any mediating effect on the relationship between the change of LUTS severity and the change of physical aspects of HRQOL, even though the previous cross-sectional study found that the association between LUTS severity and the physical aspects of generic HRQOL was partially mediated by mental health. Our findings illustrated the discrepancy in results between cross sectional and longitudinal data, and showed the main limitation of using cross-sectional data in mediation analysis. This finding reinforces the need to use longitudinal data to analyze mediation model especially in clinical studies which put emphasis on the change in patient outcomes.

## Limitations

First, the drop-out rate was high in our follow-up interviewers. Second, this study was conducted on Chinese primary care patients recruited from public-sector settings in Hong Kong, and the results may not be generalizable to secondary care or other settings where patients might have a different disease profile. Third, all outcomes were self-reported, which might have recall bias. Future studies are needed which also use objective clinical parameters to assess the severity of LUTS to help validate these findings. Fourth, normally, patient-reported outcome measures are self-completed by study participants. Using interviewer-administered method on telephone interviews might lead to some potential bias.

## Abbreviations

CI: confidence interval
Coeff: coefficient
DASS-2: Depression Anxiety and Stress Scale-21
GOPC: general out-patient clinics
HK: Hong Kong
HRQOL: health-related quality of life
IIQ-7: Incontinence Impact Questionnaire-Short Form
IPSS: International Prostate Symptom Score
LUTS: lower urinary tract symptoms
MCS: mental component summary
NAHC-CC: The Nurse and Allied Health Clinic for Continence Care
PCS: physical component summary
SD: standard deviation
SE: standard error
SF-12 v2: The Short From-12 Health Survey Version 2

## References

[1] Irwin DE, Kopp ZS, Agatep B, Milsom I, Abrams P (2011). Worldwide prevalence estimates of lower urinary tract symptoms, overactive bladder, urinary incontinence and bladder outlet obstruction. BJU Int.

[2] Wong SY, Hong A, Leung J, Kwok T, Leung PC, Woo J (2006). Lower urinary tract symptoms and depressive symptoms in elderly men. J Affect Disord.

[3] Choi EP, Lam CL, Chin WY (2016). Mental health of Chinese primary care patients with lower urinary tract symptoms. Psychol Health Med.

[4] Choi EP, Lam CL, Chin W-Y (2014). The health-related quality of life of Chinese patients with lower urinary tract symptoms in primary care. Qual Life Res.

[5] MacKinnon DP, Krull JL, Lockwood CM (2000). Equivalence of the mediation, confounding and suppression effect. Prev Sci.

[6] Richiardi L, Bellocco R, Zugna D (2013). Mediation analysis in epidemiology: methods, interpretation and bias. Int J Epidemiol.

[7] Choi EP, Lam CL, Chin WY (2015). Mental Health mediating the relationship between symptom severity and health-related quality of life in patients with lower urinary tract symptoms. Lower Urin Tract Symptoms..

[8] Chin WY, Choi EP, Wan EY, Chan AK, Chan KH, Lam CL (2017). Evaluation of the outcomes of care of nurse-led continence care clinics for Chinese patients with lower urinary tract symptoms, a 2-year prospective longitudinal study. J Adv Nurs.

[9] Choi EP, Chin WY, Lam CL, Wan EY (2015). The responsiveness of the International prostate symptom score, incontinence impact questionnaire-7 and depression, anxiety and stress scale-21 in patients with lower urinary tract symptoms. J Adv Nurs..

[10] Choi EP, Lam CL, Chin W-Y (2014). Validation of the International prostate symptom score in Chinese males and females with lower urinary tract symptoms. Health Qual Life Outcomes.

[11] Choi EP, Lam CL, Chin W-Y (2014). The Incontinence Impact Questionnaire-7 (IIQ-7) can be applicable to chinese males and females with lower urinary tract symptoms. Patient Patient Cent Outcomes Res.

[12] Lam CL, Wong CK, Lam ET, Huang W (2010). Population norm of Chinese (HK) SF-12 health survey_version 2 of Chinese adults in Hong Kong. Hong Kong Pract.

[13] Lam CL, Eileen Y, Gandek B (2005). Is the standard SF-12 health survey valid and equivalent for a Chinese population?. Qual Life Res.

[14] Lovibond SH, Lovibond PF (1996). Manual for the depression anxiety stress scales.

[15] Taouk M, Lovibond P, Laube R (2001). Psychometric properties of a Chinese version of the 21-item Depression Anxiety Stress Scales (DASS21).

[16] Baron RM, Kenny DA (1986). The moderator-mediator variable distinction in social psychological research: conceptual, strategic, and statistical considerations. J Pers Soc Psychol.

[17] Preacher KJ, Hayes AF (2008). Asymptotic and resampling strategies for assessing and comparing indirect effects in multiple mediator models. Behav Res Methods.

[18] Shrout PE, Bolger N (2002). Mediation in experimental and nonexperimental studies: new procedures and recommendations. Psychol Methods.

[19] Chin WY, Choi EPH, Wan EYF, Lam CLK (2016). Health-related quality of life mediates associations between multi-morbidity and depressive symptoms in Chinese primary care patients. Fam Pract.

[20] Glover L, Gannon K, McLoughlin J, Emberton M (2004). Men’s experiences of having lower urinary tract symptoms: factors relating to bother. BJU Int.

[21] Ashworth PD, Hagan MT (1993). The meaning of incontinence: a qualitative study of non-geriatric urinary incontinence sufferers. J Adv Nurs.

[22] Engström G, Henningsohn L, Steineck G, Leppert J (2005). Self-assessed health, sadness and happiness in relation to the total burden of symptoms from the lower urinary tract. BJU Int.

[23] Williams JM, Little MM, Scates S, Blockman N (1987). Memory complaints and abilities among depressed older adults. J Consult Clin Psychol.

[24] Maddux JE (2013). Self-efficacy, adaptation, and adjustment: theory, research, and application.

